# Simplified Bioprinting-Based 3D Cell Culture Infection Models for Virus Detection

**DOI:** 10.3390/v12111298

**Published:** 2020-11-12

**Authors:** Robert Koban, Tobias Lam, Franziska Schwarz, Lutz Kloke, Silvio Bürge, Heinz Ellerbrok, Markus Neumann

**Affiliations:** 1 Highly Pathogenic Viruses, Centre for Biological Threats and Special Pathogens, Robert Koch Institute, Seestr. 10, 13353 Berlin, Germany; robertkoban@gmx.de (R.K.); SchwarzF@rki.de (F.S.); neumannm@rki.de (M.N.); 2 Cellbricks GmbH, Gustav-Meyer-Allee 25, 13355 Berlin, Germany; tl@cellbricks.com (T.L.); lk@cellbricks.com (L.K.); 3 Advanced Light and Electron Microscopy, Centre for Biological Threats and Special Pathogens, Robert Koch Institute, Seestr. 10, 13353 Berlin, Germany; BuergeS@rki.de

**Keywords:** 3D cell cultivation, 3D bioprinting, virus infection, detection, infection model

## Abstract

Studies of virus–host interactions in vitro may be hindered by biological characteristics of conventional monolayer cell cultures that differ from in vivo infection. Three-dimensional (3D) cell cultures show more in vivo-like characteristics and may represent a promising alternative for characterisation of infections. In this study, we established easy-to-handle cell culture platforms based on bioprinted 3D matrices for virus detection and characterisation. Different cell types were cultivated on these matrices and characterised for tissue-like growth characteristics regarding cell morphology and polarisation. Cells developed an in vivo-like morphology and long-term cultivation was possible on the matrices. Cell cultures were infected with viruses which differed in host range, tissue tropism, cytopathogenicity, and genomic organisation and virus morphology. Infections were characterised on molecular and imaging level. The transparent matrix substance allowed easy optical monitoring of cells and infection even via live-cell microscopy. In conclusion, we established an enhanced, standardised, easy-to-handle bioprinted 3D-cell culture system. The infection models are suitable for sensitive monitoring and characterisation of virus–host interactions and replication of different viruses under physiologically relevant conditions. Individual cell culture models can further be combined to a multicellular array. This generates a potent diagnostic tool for propagation and characterisation of viruses from diagnostic samples.

## 1. Introduction

In vitro cell cultivation of eukaryotic cells is an established technique for virus propagation and characterisation for research and diagnostic purposes since the late 1940s [1]. However, differences between cells in conventional in vitro two-dimensional (2D) monolayer cultivation and cells in vivo may subsequently lead to altered virus–host interactions or impaired permissiveness of the cells for certain viruses. For example, *human papillomavirus* (HPV), *human norovirus* (HuNoV), and *Molluscum contagiosum virus* (MoCV) are not able to mature in 2D cultures because of inadequate cell differentiation [2,3,4]. This may cause false negative results when diagnostic samples are analysed through virus cultivation. However, also, upon successful infection, 2D cultures may produce questionable results as the altered physiology of the cells may also alter steps of virus replication compared to infection of cells in natural tissues [5]. Therefore, conventional 2D cell cultures might alter growth characteristics. In consequence, this might also alter the outcome e.g., of screening approaches for new antiviral molecules in infected 2D cultures. This in turn could affect the planning of animal experiments or the outline of clinical studies. Furthermore, it might lead to misinterpretation of the efficiency of viral inhibitors and thus might hinder the search for effective antiviral substances [6,7].

Additionally, host range or cell tropism of a virus might be altered in conventional 2D cultivation. For example, *Hepatitis E virus* (HEV) and HuNoV are unable to replicate in in vitro cultured human cells. However, they can replicate to high titres in the respective cells in vivo [8,9]. Therefore, characterisation of such a virus in 2D cultures can lead to misinterpretation of host range, tissue specificity, or pathogenicity and thus in consequence affect the risk assessment of a pathogen. Moreover, 2D cultures are often not stable for an extended period of time, which may hinder propagation of slowly replicating viruses such as *Hepatitis A virus* (HAV) or *Puumala virus* (PUUV). In consequence, it might preclude propagation of such viruses from diagnostic samples when initial titres are too low for successful replication [10,11]. 2D cultures usually consist of only one cell type. In order to cultivate an unidentified virus from a diagnostic sample several cell lines might have to be tried for virus propagation, provided that sufficient sample material is available.

3D cultivation of cells may overcome these limitations of 2D cell cultures. 3D cultures can comprise one or several cell types which are embedded in a matrix similar to the extracellular matrix of tissues in vivo. The more in vivo-like biology of cells in 3D cultures may enable mimicry of biologically relevant phenomena [12]. This might also be relevant for virus replication since fully differentiated 3D cultures of human skin analogues and other tissue analogues enabled in vitro propagation of viruses such as HPV, HuNoV, and *adeno-associated virus* type *2 (AAV2)*, that could not be cultivated with conventional cultivation techniques [8,13,14]. Other studies revealed that cells in virus-infected 3D cultures behave similarly to the infected cells in native tissues and thus allow more precise insights into biological processes in vivo during infection than conventional 2D cultures [3,15,16].

Experiments with candidate substances for antiviral treatment revealed that cultivation of cells either in 3D or conventional 2D cultures may affect selectivity and/or efficacy of an antiviral drug which in consequence may lead to an altered assessment of its antiviral potential for in vivo application [6,7]. Moreover, the use of microfluidic organ chips could enable the application of different organotypic models in one cocultivation approach for specific questions in basic virus research e.g., cross-talk between infected and uninfected cells in tissues [17].

Despite obvious advantages of 3D cell cultures over conventional monolayers, the former also entail considerable technical problems that may compromise their use in routine procedures, particularly in diagnostics. Additionally, for most 3D cell culture models, with the exception of simple spheroid cultures, it is almost impossible or associated with very high costs to set up high-throughput approaches. This is particularly problematic for drug screenings [18]. A special limitation of 3D cultures is their lack of transparency. This impedes microscopy and the live screening of cell growth, which is a basic and simple advantage in conventional 2D cultures that allows tracking of virus infection e.g., through the observation of cytopathic effects (CPE) and cell fusion events.

A promising and aspiring option to circumvent these limitations is represented by 3D bioprinting [19]. This technology allows to print components of biological tissues, such as extracellular matrices as a scaffold that can be seeded with cells, or to generate whole tissue equivalents, including the respective cell types in their in vivo typical arrangement [20,21]. One of the major advantages of this method is the possibility to precisely define composition and arrangement of a culture. Therefore, the design and morphological properties of a matrix can be freely defined and thus be optimised for practical use, even for high-throughput applications [22]. In recent years, bioprinted 3D cultures have already been applied in the field of toxicology and in liver disease modelling, where bioprinted tissue constructs helped to prolong cultivation times, thus giving detailed insights into safety and efficacy testing [23].

Furthermore, bioprinting technologies are increasingly applied to the development of biomedical devices and to tissue engineering in general [24]. Advances in bioink preparation including the incorporation of decellularised matrices and induced pluripotent stem cells open up broad applications in diagnostics and regenerative medicine [25]. Additionally, it was recently shown that cells behave more in vivo-like within 3D bioprinted cell cultures during *influenza A virus* infection than in a corresponding infected 2D culture [26].

The aim of this study was to establish 3D bioprinting-based 3D cell culture models for infection studies with a broad range of viruses that are easy to handle, rapidly modifiable, and definable according to the requirements of the experiment. The infection models should help basic research to explore in vivo-like virus replication and host response as well as the detection of pathogenic viruses. It should also serve as a convenient tool for propagation of viruses which (i) cannot replicate in 2D cultures, (ii) replicate too slowly to be cultivated in 2D cultures, or (iii) originate from diagnostic samples with titres too low for successful replication in 2D cultures. As a proof of concept, five model viruses with different characteristics regarding host range, cell tropism, genome characteristics, cytopathogenicity, and replication rate were used as infectious agents. They were combined with five cell culture models, established from five different cell types with different host and tissue origin covering a wide spectrum of possible virus–host–tissue combinations.

## 2. Materials and Methods

### 2.1. Cells and Culture Conditions

All cell lines applied are listed in Table A1. Cells were adapted to a uniform growth medium consisting of DMEM with 10% foetal bovine serum (FBS; Biochrom, Berlin, Germany, DE), 2 mM L-Glutamine (Thermo Fisher Scientific, Waltham, MA, USA), and 1X MEM nonessential amino acids (Thermo Fisher Scientific) for several passages prior to application to 3D culture generation. Cells were cultivated at 37 °C and 5% CO_2_ in a humidified incubator (later referred to as standard conditions).

### 2.2. 3D Bioprinting of Cultivation Matrices

**Figure 1:**
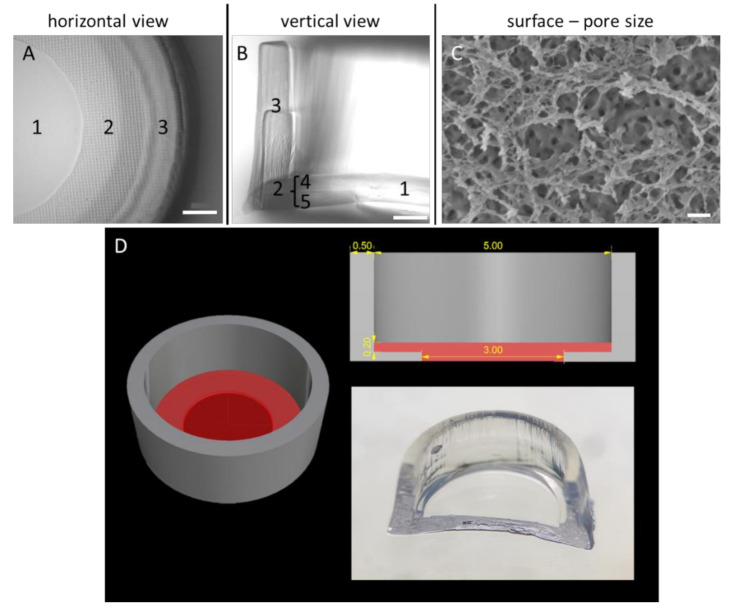
Morphological and structural characterisation of the Wellbrick matrix. (**A**) Partial microscopic view onto the matrix surface. The growth area of the Wellbrick is divided into (1) a “viewing window” for light microscopy consisting of gelatine only and (2) a hybrid area consisting of gelatine supported by a layer of polyethylene glycol (PEG). The boundary of the Wellbrick (3) consists of PEG only. (**B**) Vertical cut through a Wellbrick. The outer PEG boundary (3) is printed in two layers on top of each other. The hybrid growth area is separated into two equal parts of an upper gelatine layer (4) supported by a layer of PEG (5) as physical support. (**C**) Cryo-scanning electron micrograph of partially freeze-dried gelatine surface. The surface layer was completely dried and reveals a fine mesh-like structure which is partially destroyed by the freeze-drying process. Below this layer, a surface with distinct pores is visible which are most likely surrounded by fully hydrated, frozen gelatine. Scale bar (**A**,**B**)= 500 µm. Scale bar (**C**) = 200 nm. (**D**) Dimensions and shape of Wellbricks model. The photography shows a cut through the centre of a Wellbrick. The length specifications on the CAD image at the top right are indicated in millimetres.

Cultivation scaffolds were designed using appropriate CAD software (Rhinoceros 5; McNeel Europe, Barcelona, Spain, ES). Afterwards, the 3D model (Figure 1D) was printed layer by layer using a stereolithographic bioprinter (Cellbricks GmbH, Berlin, Germany, DE) capable of printing multiple materials within one printing process [21]. Up to 24 scaffolds are printed simultaneously. The optimised specimen, which was named Wellbrick, consists of PEGDA700 (Sigma-Aldrich, St. Louis, MO, USA) for the wall and main ring structure of the bottom and methacrylated gelatine (GelMA) as the cultivation matrix with a central window without PEGDA700 support for microscopy (Figure 1A,D in red). The rim of the cultivation matrix was integrated into the PEGDA wall to ensure structural integrity.

GelMA was synthesised as previously described [27,28]. In short, 10 wt% gelatine (porcine skin Type A, 300 bloom; Sigma-Aldrich) was dissolved in phosphate buffered saline (PBS; Thermo Fisher Scientific) at 50 °C. Then, methacrylic anhydride (Sigma-Aldrich) was added and the reaction continued for 2 h. After the reaction, the product (GelMA) was purified and freeze dried. In this study, we compared scaffolds in four configurations. All scaffolds consist of 10% PEGDA700. For the cultivation area, GelMA at 7 wt% (compression modulus E_k_ = 11.9 kPA) or 10 wt% (E_k_ 16.4 kPA) solved in culture medium was used. For half of the scaffolds, Collagen A solution (Biochrom, Berlin, Germany, DE) was added to the bioink at 3%. For all four conditions, Lithium phenyl-2,4,6-trimethylbenzoylphosphinate was used at 0.1 wt% as photoinitiator [29]. After printing, the scaffolds were washed and stored in PBS at 4 °C until further use ready for cell seeding procedures.

### 2.3. Generation of 3D Cell Cultures on the Wellbricks Matrices

Wellbricks were transferred with coverglass tweezers from the storage medium to a new 24-well cell culture plate (Thermo Fisher Scientific) and were washed with 1 mL of PBS. PBS was removed from the plates and replaced by 1 mL of growth medium. Wellbricks were equilibrated at standard conditions for at least 24 h and were washed with 1 mL of growth medium to remove residual storage medium components. For cell seeding, cells grown to 70–80% confluency in standard T-75 cell culture flasks (Thermo Fisher Scientific) were washed twice with 20 mL of PBS and then were detached using 2 mL of Trypsin-EDTA (Thermo Fisher Scientific) at standard conditions for 5 min. Detachment was stopped by adding 8 mL of growth medium followed by centrifugation (300× *g*, 5 min). Cell pellets were suspended in 5 mL of growth medium and cell counts were determined. Appropriate volumes of the cell suspensions were diluted with growth medium to reach defined seeding concentrations (BHK-21: 2.3 × 10^5^/mL; CRFK: 7 × 10^5^/mL; HeLa: 9.3 × 10^5^/mL; HMEC-1: 10.5 × 10^5^/mL; Vero E6: 4.7 × 10^5^/mL). Concentrations were optimised in a previous study (data not published) to enable fully confluent 3D cultures of the different cell lines approximately at the same time point 2 days post cell seeding.

Growth medium was completely removed from the plate wells and Wellbricks were centred within the wells using a pipette tip. Growth medium was then carefully removed from the Wellbricks’ reservoirs which then were filled with 30 μL of the respective cell suspension. To prevent drying of the Wellbricks, 200 µL of growth medium was added to the outer area of the specimens. After complete cell adhesion, 800 μL of growth medium was carefully added via the borders of the plate wells 5 h post cell seeding. Cells were either cultured for 7 days as uninfected controls or were infected with the different viruses 3 days after seeding (see Section 2.5). Half of the medium was exchanged by fresh growth medium every 3 days for sufficient nutrient supply and to maintain cellular environment.

### 2.4. Virus Propagation, Purification, and Titration

Recombinant *Cowpox virus* strain Brighton Red (CPXV BR) BAC pBRf consists of the full-length CPXV BR (ATCC, #VR-302) genome with a mini-F sequence and a GFP gene integrated into the thymidine kinase locus of the viral DNA [30]. Preparation of virus stocks and virus titration was performed as described previously [6].

*Feline calicivirus* (FCV; Robert Koch Institute isolate, Georg Pauli) was propagated in CRFK cells. FCV stock was diluted 1:250 in 10 mL of growth medium and added to the cells cultivated in T-175 flasks (Thermo Fisher Scientific). After 1 h of incubation under standard conditions, 20 mL of growth medium was added to the infected cells. Then, 24 h post infection supernatant was harvested, centrifuged (1000× *g*, 10 min, 4 °C), and finally stored at −80 °C.

*Modified Vaccinia Ankara* (MVA; BN-MVA 070602, Bavarian Nordic, Planegg, Germany, DE) was propagated in BHK-21 cells. MVA stock was diluted 1:50 in 10 mL of growth medium and added to the cells cultivated in T-175 flasks. After 2 h of incubation under standard conditions, 20 mL of growth medium was added, and infected cells were incubated for 5 more days. Afterwards, cells were harvested with a cell scraper and were centrifuged (1000× *g*, 10 min, 4 °C). Cell pellets were suspended in 2 mL of growth medium and were disrupted by three cycles of freezing at −80 °C and thawing at 37 °C and subsequent sonication with a Bioruptor^®^ Plus (Diagenode; Denville, NJ, USA) at high intensity (three cycles: 30 s/30 s pause, 4 °C). Cell suspensions were centrifuged (1000× *g*, 5 min, 4 °C) and supernatant was stored at −80 °C.

*Puumala virus Kazan* (PUUV Kazan; Robert Koch Institute isolate, Matthias Niedrig) propagation was performed in Vero E6 cells. 500 µL of PUUV stock was diluted 1:4 in infection medium (DMEM, 2 mM L-Glutamine, 1% FBS) and was added to a decanted T-175 flask of Vero E6 cells. After 2 h, 28 mL of growth medium (DMEM, 2 mM L-Glutamine, 5% FBS) were added to the cells which were then cultivated for 14 days under standard conditions (addition of fresh 10 mL of growth medium after 6 days). After incubation, 30 mL of supernatant was decanted and cell culture flasks with remaining medium were frozen at −20 °C and thawed at room temperature (RT) (three cycles). Cells were harvested with a cell scraper and suspensions were sonicated (three cycles: 30 s/30 s pause, 4 °C). Cell suspensions were centrifuged (1000× *g*, 5 min, 4 °C) and supernatant was stored at −80 °C.

For propagation of *Yellow Fever virus* vaccine strain 17D (YFV 17D; stamaril, Sanofi Pasteur, Lyon, France, FR), Vero E6 cells cultured in T-175 flasks were detached as described in Section 2.3 and were suspended in 29 mL of growth medium with 1.6 mL of YFV 17D stock. Suspension was placed in a new T175 flask and incubated under standard conditions for 4 days. After incubation, supernatants were centrifuged (5 min, 1000× *g*, 4 °C) to pellet cell debris and were stored at −80 °C.

Viruses were purified by ultracentrifugation with an Optima XPN-100 (Beckman Coulter; Brea, CA, USA) through different sucrose cushions and under specific conditions according to Table A2. After centrifugation, virus pellets were suspended with 1 mL of 10 mM TrisHCl and stored at −80 °C. Resuspended PUUV pellets were further sonicated (three cycles: 30 s/30 s pause, 4 °C) before freezing.

For PUUV and MVA, quantification of infectious viruses was performed by the focus-forming unit (FFU) assay adapted from a previously described protocol [31]. For YFV and FCV, quantification was performed by 50% tissue culture infectious dose (TCID50) assay adapted from a previously described protocol [32]. Titration of PUUV and YFV was performed on Vero E6 cells. FCV titre was determined on CRFK cells and MVA titre on BHK-21 cells. For PUUV, FCV, and MVA titration cells were preincubated in black optical-bottom 96-well plates (Thermo Fisher Scientific) for 24 h and for YFV titration 1 h prior to infection. Decadal dilutions of virus stocks were added to the cells, and cells were covered with medium containing carboxymethyl cellulose to a final concentration of 1.6% 2 h post infection. FCV-infected cells were incubated for 2 days, MVA-infected cells for 3 days, YFV-infected cells for 6 days, and PUUV-infected cells for 7 days. After incubation the cells were washed with PBS, fixed with ice-cold methanol, and foci of infected cells were detected using following virus-specific antibodies diluted 1:500 in PBS with 2% BSA and 0.2% NaN3: anti-hantavirus nucleocapsid protein (Abcam, Cambridge, United Kingdom; ab34757, mouse), anti-Vaccinia Virus Lister Strain (OriGene, Rockville, MD, USA; BP1076, rabbit), anti-FCV 1-43 (Santa Cruz Biotechnology, Dallas, TX, USA; sc-80785, mouse), and mouse anti-YFV 6330 [33]. Antigen–antibody complexes were detected with AF488-conjugated anti-mouse (Cell Signaling Technology, Cambridge, United Kingdom; #4408, 1/1000) and AF488-conjugated anti-rabbit (Cell Signaling Technology, Cambridge, United Kingdom; #4412, 1/1000) antibodies, respectively. Fluorescence of infected cells was detected using a fluorescence microscope. All samples were tested in quadruplicate. All virus stocks were screened for absence of mycoplasma contamination by qPCR [34].

### 2.5. Infection of 3D Cell Cultures

For infection of 3D cultures, growth medium was completely removed from the plate wells and Wellbricks were centred within the wells using a pipette tip 3 days post cell seeding. Infection parameters had been optimised in a previous study with regard to reaching comparable infection rates of the different viruses to defined time points. Based on this, virus suspensions with defined infectious units (IU) were prepared in an appropriate volume of growth medium. CPXV and FCV were diluted to 3.3 × 10^3^ IU per mL, MVA to 6.6 × 10^3^ IU per mL, PUUV to 100 × 10^3^ IU per mL, and YFV to 50 × 10^3^ IU per mL. Growth medium was then carefully removed from the reservoirs of the Wellbricks which were then filled with 30 μL of the respective virus suspension. To prevent drying of the Wellbricks, 200 µL of growth medium was added to the outer area of the specimens. After 3 h, 800 μL of growth medium was carefully added via the borders of the plate wells to provide sufficient media supply. Cells were cultured for 24 h (FCV), 48 h (CPXV), 72 h (MVA and YFV), or 96 h (PUUV) under standard conditions. Half of the medium was exchanged by fresh growth medium every 48 h for sufficient nutrient supply and to maintain cellular environment.

### 2.6. Cryo-Scanning Electron Microscopy (Cryo-SEM) of the Wellbrick Surface

A cell-free Wellbrick was incubated in fixative (2.5% glutaraldehyde, 1% formaldehyde in 0.05 M HEPES buffer) for 30 min at room temperature, followed by ethanol/water mixtures (30/50%). To prepare for freezing, sample regions were excised with a biopsy punch (Ø 2 mm) and transferred into the recess of an aluminium sample carrier (Ø 3 mm, height 0.5 mm, recess 0.3 mm; Engineering Office M. Wohlwend GmbH, Sennwald, Switzerland, CH) originally used for high-pressure freezing. The sample was frozen by placing the aluminium carriers on a metal plate which was cooled with liquid nitrogen (lN_2_). The frozen sample was mounted on an LN_2_-cooled sample holder (freeze-fracture clamp holder for 3 mm carriers, No. 16LZ04746VN, Leica Microsystems, Wetzlar, Germany, DE) and transferred into a precooled (−140 °C) cryo-sputter coater (MED 020; Leica Microsystems). To remove the ethanol/water mixture from the surface layer of the sample, the sample holder was heated to −100 °C for 5 min at 1 × 10^−5^ to 4 × 10^−6^ mbar. Finally, the sample was sputter-coated with 2 nm of platinum at −120 °C and transferred to the precooled scanning electron microscope (LEO 1530 Scanning Electron Microscope; Carl Zeiss Microscopy, Jena, Germany, DE). Cryo-transfer was carried out using the Leica VCT system (VCT 100; Leica Microsystems). The samples were examined at −120 °C stage temperature and an accelerating voltage of 3 kV (working distance = 5–6 mm), using the in-lens secondary electron detector.

### 2.7. Immunofluorescence Assay (IFA)

For IFA analysis, growth medium of the culture plates was removed, and 3D cultures were washed with 1 mL of PBS. Cultures were fixed with 500 µL of 4% paraformaldehyde for 1 h at RT. After successive dehydration with 500 µL of 15% and 30% sucrose for 2 h each, 3D cultures were embedded in 400 µL of Tissue-Tek (Sakura, Staufen, Germany, DE) in sample vials and equilibrated for 2 h at RT. After flash-freezing in liquid nitrogen, specimens were stored at −80 °C. Frozen sections of 8 µm thickness were prepared with the cryomicrotome Leica CM1950 (Leica Microsystems) and transferred to SuperFrost^®^ Plus Gold adhesion microscope slides (Thermo Fisher Scientific).

Immunological staining of the sections and monolayer cultures including staining of cell membranes with WGA-CF594 (Biotium, Fremont, CA, USA) was performed as described previously [6]. Cellular proteins were stained with antibodies against integrin beta 1 (Abcam, Cambridge, UK; #30388, mouse), atypical protein kinase C (Santa Cruz Biotechnology, Dallas, TX, USA; sc-208, rabbit), and Collagen IV (Abcam, Cambridge, UK; #6586, rabbit) diluted at 1/100 in PBS with 2% BSA and 0.2% NaN_3_. Virus proteins were stained with the antibodies listed in Section 2.4. Antigen–antibody complexes were detected with AF647-conjugated anti-mouse (Cell Signaling Technology, Cambridge, UK; #4410, 1/500) or AF647-conjugated anti-rabbit (Cell Signaling Technology, Cambridge, UK; #4414, 1/500) antibodies. CPXV-infected cells were visualised by monitoring GFP expression from the recombinant CPXV strain. Counterstaining of nuclei and mounting was performed with Vectorshield mounting medium (Thermo Fisher Scientific).

Digital vertical sections of monolayer cultures were generated by overlay of 30–35 horizontal microscopic records which were shifted vertically in 300-nm steps. Multichannel fluorescence images and digital vertical sections were taken with the confocal laser scanning microscope LSM 780 (Carl Zeiss Microscopy). Analysis and processing of raw microscopy data were performed by using the software Zen 2012 (blue edition SP2, Carl Zeiss Microscopy).

### 2.8. Live Cell Imaging of Infected 3D Cultures

Wellbricks were seeded with Vero E6 cells (see Section 2.3) and infected with CPXV BR (see Section 2.5) in standard growth medium without phenol red and with additional 1X MEM Vitamin Solution (Thermo Fisher Scientific). Then, 4 h post infection, cell cultures were transferred to an automated inverted microscope (Nikon Eclipse TE2000-E; Nikon Corp., Tokyo, Japan, JP) equipped with an incubation chamber with temperature and CO_2_ control (Solent Scientific Ltd.; Portsmouth, UK) and a Xenon illumination system (DG4; Sutter Instrument Company, Novato, CA, USA) for epi-fluorescence live cell imaging.

Cells were examined using a 20 × 0.5 NA Plan Fluor lens (Nikon Corp.), and images were captured with an EMCCD (512 × 512 pixel, 16 bit, Cascade II 512; Roper Scientific, Martinsried, Germany, DE). Imaging of GFP fluorescence was done with an excitation wavelength of 485 nm, and emission was detected with a DAPI/FITC/TxRed tripleband filter.

The infected cells were observed for 48 h and images were taken automatically every 20 min in fluorescence mode and in bright field mode. Cells were cultivated at standard conditions for the duration of the experiment. Image processing was performed with Fiji (version Madion) [35].

### 2.9. DNA/RNA Preparation and Real-Time PCR Assays

For preparation of nucleic acids Wellbricks were washed with PBS, harvested in 400 μL of RLT buffer (RNeasy Mini Kit; Qiagen, Hilden, Germany, DE) supplemented with 1% 2-mercaptoethanol in 1.5-mL reaction tubes and stored at −80 °C. DNA/RNA purification was performed as previously described [6]. For DNA viruses, purified nucleic acids were used for qPCR analysis. For RNA viruses, reverse transcription of purified nucleic acids was performed with random hexamer primers and MultiScribe™ MuLV reverse transcriptase (Invitrogen, Carlsbad, CA, USA) according to standard protocols.

Quantification of viral genome equivalents (GE) was performed by real-time PCR (qPCR) and normalised to the human reference gene *MYC* (NM_002467) as previously described [6]. GE of CPXV and MVA were quantified by determination of the *CPXV086* gene (AF482758, nomenclature CPXV BR) with previously published primers and probes [6]. Quantification of PUUV GE was performed by determination of the nucleocapsid protein (N) gene [36]. GE of YFV were quantified by determination of the 17D polyprotein gene (JX949181.1; forward: 5′-TgTCAggggAgCTAggAgAA-3′; reverse: 5′-gAAgCCCAAgATggAATCAA-3′; probe: 5′-FAM-CgAggAgTgggAgTggCACA-BHQ1-3′).

Quantification of FCV was performed by a qPCR assay targeting the viral genome-linked protein gene in *ORF1* (NC_001481.2; forward: 5′-ACCATACAgAggTCgTggTg-3′; reverse: 5′-CTCgACCgACAAgTCCAA-3′; probe: 5′-FAM-CATCATATTCATCATCCgTgAgAgCCA-BHQ1-3′).

### 2.10. Cell Viability Assay

Cell viability was determined with the ATP-based CellTiter-Glo^®^ luminescent cell viability assay (Promega, Madison, WI, USA). Viability of monolayer cultures was analysed according to the manufacturer’s protocol but homogenisation time on an orbital shaker was extended from 2 to 10 min for efficient cell lysis. For 3D cultures, specimens were washed with 500 µL of PBS and were transferred with coverglass tweezers to standard 1.5-mL reaction tubes containing 110 µL of fresh growth medium. A total of 110 µL of the CellTiter-Glo^®^ reagent was added and 3D cultures were homogenised on an orbital shaker at 700 rpm for 20 min at RT. After additional incubation of 10 min at RT 220 µL of fresh medium was added to the samples and 200 µL of each sample was transferred into two wells of a 96-well white polystyrene microplate (Corning, Corning, NY, USA). Luminescence was measured on a Tecan Infinite plate reader with i-control software (Tecan, Männedorf, Switzerland, CH).

## 3. Results

### 3.1. 3D Bioprinted Matrices Are Structurally Suitable for an Easy-to-Handle 3D Cell Cultivation

Prior to the establishment of the 3D cell culture infection models, a suitable matrix had to be designed as an appropriate support for 3D cultivation. On the one hand, the matrix had to fulfil specific requirements for a stable and controlled handling and, on the other hand, it had to possess structural properties that are also inherent to biological extracellular matrices.

To optimise the handling and user friendliness of the specimens, the format of the matrices was developed via an iterative process whereby a matrix format with distinct morphological characteristics could be established which was suitable for high-throughput approaches in 96-well cell culture plates. The integration of a chemically inert, cell-repellent boundary of the actual gelatine growth surface consisting of PEGDA (Figure 1B) enabled a focused and therefore efficient cell application to the matrices. In the peripheral areas of the growth surface, where PEGDA is imprinted under the GelMA layer as a stabilisation element, microscopy of cells growing on the matrix was hampered because of the pronounced PEGDA grid (Figure 1A). However, through a central recess of the PEGDA supporting layer underneath the GelMA growth surface, microscopy of cells growing in this central “viewing window” was possible. This combined structure consisting of the GelMA matrix and partial PEGDA support for enhanced stability of the cultivation area was developed to allow on one hand microscopic observation of a significant part of the culture surface and on the other hand to generate sufficient material for biochemical and molecular analyses. The optimised specimen was named Wellbrick.

To characterise the topography of the GelMA matrix, the surface structure of the growth area of the Wellbricks was analysed by SEM (Figure 1C). It was observed that the upper layer of Wellbricks, consisting of 10 wt% GelMA without Collagen A addition, had a pore diameter of approx. 50–200 nm. This pore size corresponds to that of biological basal laminas and extracellular matrices, suggesting a general suitability of the matrices for 3D cultivation.

### 3.2. Matrix Composition Influences Viability of the Wellbricks Cell Cultures

In order to provide a broad spectrum of potential host cells for different human and animal pathogenic viruses for research and detection, 3D cell culture models with five cell lines were established. These cell lines originate from three different tissues, four different species, and represent three distinct cell types (Table A1). Cell growth and cell viability on hydrogels, which were also used in this study, are strongly dependent on the matrix structure and composition [37]. Since this dependence for 3D growth had already been observed for primary human epithelial cells (data not shown), the growth of the five cell lines was tested on different matrix variations with either high (10 wt%) or low (7 wt%) GelMA concentrations. Furthermore, the addition of native collagen in the upper layer of the matrix was examined as a representative for a basal lamina.

**Figure 2:**
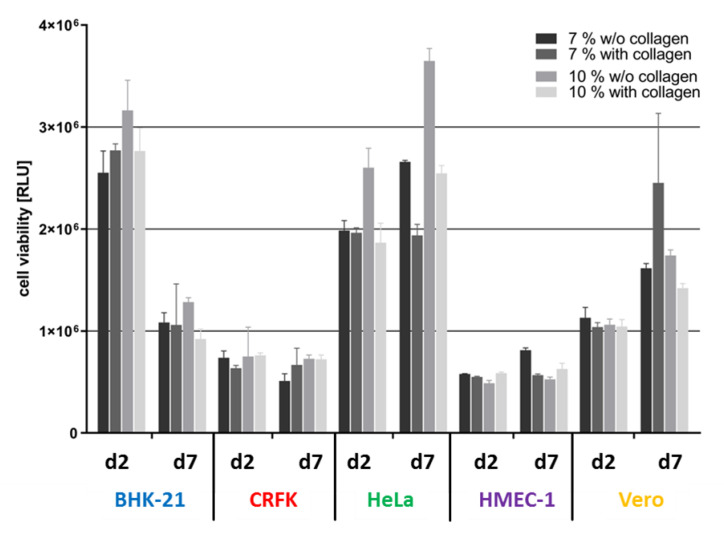
Influence of the Wellbrick matrix composition on the viability of different cell lines. BHK-21, CRFK, HeLa, HMEC-1, and Vero cells were seeded on Wellbricks. Cultivated cells reached confluence on day 2 and were cultivated for an additional 5 days to investigate long-term effects on viability. Cells were either grown on 7 or 10 wt% gelatine with or without 3% Collagen A supplement. Cell viability of 3D cultures was determined by ATP measurement with the CellTiter-Glo^®^ cell viability assay.

A potential effect of these variations on the biology of the different cells was determined at an early and a late time point after cell seeding (Figure 2). For CRFK and HMEC-1 cells neither matrix composition nor cultivation time had an influence on cell viability. Viability of Vero cells increased with time on all matrices and showed the highest viability on matrices composed of 7 wt% gelatine with collagen. BHK-21 and HeLa cells had the highest viability on a 10 wt% gelatine matrix without collagen at both time points investigated. In an earlier study, it had also been shown that primary human epithelial cells had a significantly higher viability on matrices consisting of 10 wt% gelatine without collagen than on matrices with other compositions. These data suggest that the 10 wt% gelatine without collagen composition yielded a matrix that best supported the growth of all cell lines tested and therefore was applied in all further experiments in the course of this study.

### 3.3. Cell lines Cultured on Wellbricks Show Distinct 3D-Typical Growth Characteristics

**Figure 3:**
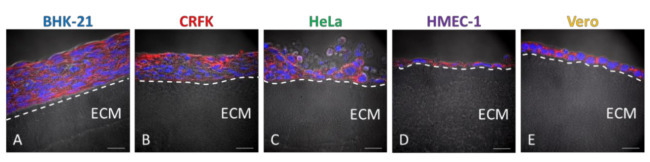
Morphological characterisation of different cell lines grown on Wellbricks. BHK-21 (**A**), CRFK (**B**), HeLa (**C**), HMEC-1 (**D**), and Vero (**E**) cells were cultivated on Wellbricks with the defined optimal matrix composition (10 wt% gelatine, w/o Collagen A) for 7 days. The 8 µm cryosections of the specimens were analysed by microscopy. Sections were treated with CF594 (red)-coupled wheat germ agglutinin (WGA) for staining of sialic acids and N-acetylglucosamin on cell membranes. Cellular nuclei were counterstained with DAPI (blue). Matrix structure (beneath the layer(s) of cells) was visualised in the brightfield channel. Scale bar = 20 µm. (**A**–**E**) The border between matrix and cell layer has been visualised with a dashed line and extracellular matrix (ECM) has been marked for ease of orientation.

To further characterise the cells grown on the optimised Wellbricks, cell morphology and localisation of in vivo relevant polarisation markers were investigated and compared to the corresponding monolayer cultures. Therefore, cell lines were grown on Wellbricks for 7 days, for each specimen vertical cryosections were prepared and stained for nuclei and plasma membrane components. Microscopic analysis of cryosections revealed that the fibroblast cell line BHK-21 as well as the epithelial CRFK and HeLa cells grew on the Wellbricks matrix in several cell layers typical of 3D cell cultures. Cells in the lower cell layers did not exhibit a spherical morphology characteristic for apoptotic cells (Figure 3A–C). In contrast, endothelial HMEC-1 cells as well as epithelial Vero cells both formed a continuous single cell layer (Figure 3D,E) which for HMEC-1 corresponds with the in vivo arrangement of the microvascular endothelium [38]. In addition, HMEC-1 cells and BHK-21 cells were morphologically much more elongated and flattened than all other cell lines which varied from round to cubic in shape and thus were uniformly expanded in all spatial dimensions. These morphological properties also correspond with the respective cell shape in vivo.

**Figure 4:**
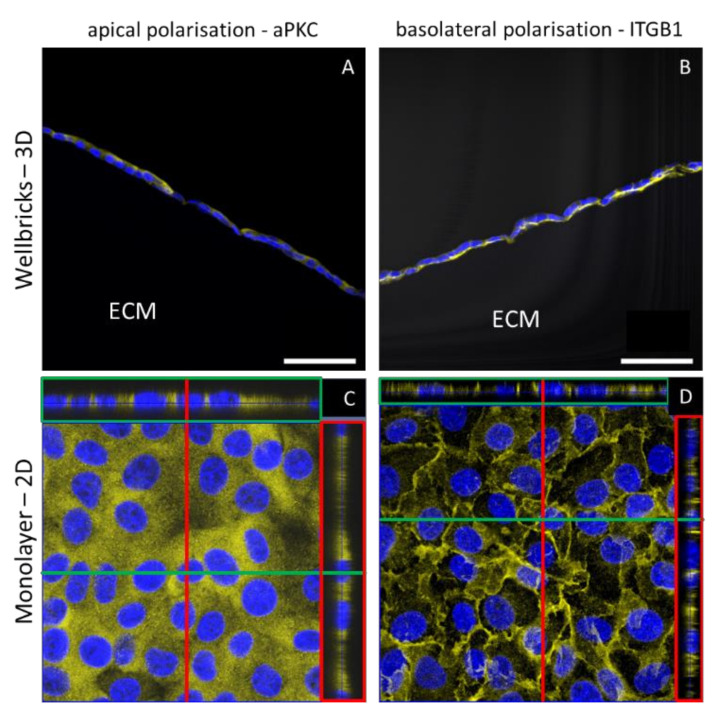
Localisation of in vivo epithelial polarisation markers in three-dimensional (3D) and two-dimensional (2D) culture of Vero E6 cells. Cells were either grown on Wellbricks (3D) or on conventional polystyren plates (2D) for 7 days. The 8 µm cryosections were prepared for 3D cultures. After permeabilisation the atypical protein kinase C (aPKC) as apical marker (**A,C**) and integrin beta 1 (ITGB1) as basal marker (**B,D**) were visualised with polyclonal antibodies and AF647 (yellow) coupled anti-rabbit secondary antibodies. Cellular nuclei were counterstained with DAPI (blue). Digital sections of 2D cultures in z-direction were generated by overlay of 30 single frames (**C**,**D**) and are shown on the right of each picture in red boxes and on top of each picture in green boxes. Scale bar = 50 µm.

In vertical cryosections of Vero cells grown on the Wellbricks matrix, the localisation of two in vivo epithelial polarisation markers was analysed via IHC and confocal scanning laser microscopy. The basal polarisation marker integrin beta 1 was localised towards the matrix, whereas the apical polarisation marker atypical protein kinase C showed an apical localisation towards the cell culture medium (Figure 4A,B). In contrast, analysis of digital vertical cryosections of 2D monolayer cultures revealed a homogenous distribution of both marker proteins over the entire depth of the cells and thus without distinct polarisation (Figure 4C,D). In addition, Vero cells grown on Wellbricks for 7 days synthesised large amounts of collagen IV, which was localised towards the Wellbricks matrix and is representative for basal lamina formation. These findings support an in vivo-like growth of the cells cultured on the Wellbrick support. Corresponding monolayer cultures, however, showed no comparable collagen IV deposition.

### 3.4. 3D-Cultured Cell Lines on Wellbricks Show Differential Permissiveness for the Infection with Different Viruses

Once the in vivo-like growth of the cell lines on the Wellbricks matrix was verified, the suitability of these cell cultures as infection models was investigated. A selection of viruses was compiled to represent a range of viral pathogens, diverse in terms of morphological and molecular characteristics as well as host range.

Individual 3D cell cultures of the five cell lines, precultivated on Wellbricks for 3 days, were each infected with a recombinant GFP-expressing CPXV strain, an FCV strain, PUUV Kazan, MVA BN, and YFV 17D. MOI and cultivation time for each virus were selected based on previous results to achieve an infection rate of 20–50% of cells at the time of analysis. Experiments were performed in triplicate. One specimen of each virus–cell combination was prepared for histological analyses and two specimens were prepared as duplicates for analyses with virus-specific qPCRs.

**Figure 5:**
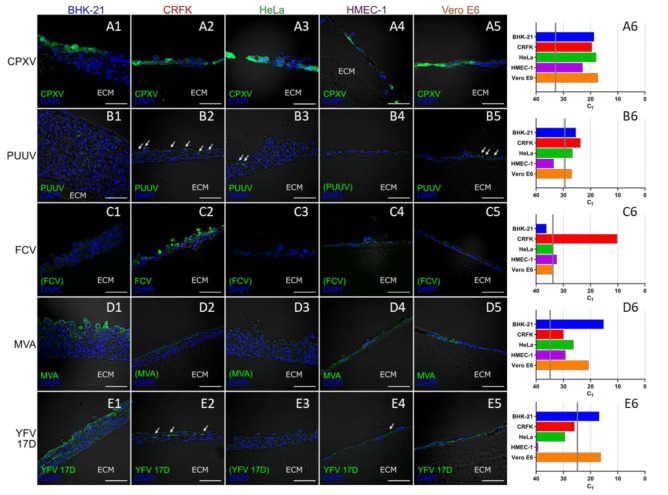
Permissiveness of different cell lines cultured on Wellbricks for infection with different viruses. The cells were cultivated on Wellbricks (10 wt% gelatine, w/o Collagen A) for 3 days and subsequently infected either with *Cowpox virus* (CPXV ) for 48 h (**A1**–**A6**), *Puumala virus* (PUUV) Kazan for 96 h (**B1**–**B6**), *Feline calicivirus* (FCV) for 24 h (**C1**–**C6**), *Modified Vaccinia Ankara* (MVA) for 72 h (**D1**–**D6**), and *Yellow Fever virus* (YFV) 17D 72 h (**E1**–**E6**). The 8-µm cryosections of the specimens were analysed by fluorescence microscopy (1–5). After permeabilisation, infected cells were visualised with virus-specific antibodies and AF647-coupled anti-rabbit or anti-mouse secondary antibodies (green) (**B1**–**B6**)**–**(**E1**–**E6**). CPXV-infected cells were visualised by monitoring GFP expression (green) from recombinant CPXV (**A1**–**A6**). Cellular nuclei were counterstained with DAPI (blue). White arrows indicate elusive spots of infected cells, scale bar = 50 µm. The micrographs shown are representative for data from several sections analysed. Average cycle threshold (C_T_) values for virus genome equivalents in cell lysates of infected cells (*n =* 2) were determined by virus-specific qPCRs (6). C_T_ values corresponding to the respective inocula are indicated as vertical grey lines.

Vertical cryosections, each immunologically stained with antibodies specific for the corresponding virus, showed infection patterns characteristic for the respective viruses. CPXV has the broadest host range of all viruses in the experiment. Accordingly, all cell lines were infected with CPXV in a comparable manner 48 h post infection (p.i.) (Figure 5(A1–A5)). Similarly, infection of all cells with the exception of HMEC-1 was observed for PUUV Kazan 96 h p.i., whereas the number of infected cells in these cultures was considerably lower than for CPXV (Figure 5(B1–B5)) despite the distinctly longer cultivation time and the considerably higher multiplicity of infection (MOI) used for PUUV infections. These results were also reflected in the quantification with virus-specific qPCR assays (Figure 5(A6,B6)). The cat-specific FCV had already infected the majority of cells in CRFK 3D cultures 24 h p.i., but was unable to replicate in any other cell line (Figure 5(C1–C6)). Both MVA and YFV 17D replicated best in BHK-21 and in Vero cells 72 h p.i., as shown by IFA (Figure 5(D1–D5) and Figure 5(E1–E5)) and qPCR analyses (Figure 5(D6,E6)). In all other cell lines replication of MVA and YFV 17D could not be detected by IFA. While MVA replication nevertheless could be confirmed by qPCR analyses also in these cell cultures. In human HeLa and HMEC-1 cells YFV 17D qPCR signals did not exceed the inoculum indicating the absence of YFV replication. BHK-21 and Vero cells were the most effective host cells for all viruses except for FCV which was specific for the cat cells. The human HeLa and HMEC-1 cells were overall the least permissive cell lines for the set of viruses tested. It was remarkable, that for the multi-layered BHK-21 and CRFK 3D cell cultures IFA results indicated that in the course of infection always only the upper layers of were infected and that the infection was unable to penetrate into the lower layers. The only exception to this was PUUV, which could be detected in BHK-21 cells almost over the entire depth of the layers of the 3D culture (Figure 5(B1)).

### 3.5. Screening of Infection in Wellbricks Cultures Is Simplified Compared to Other 3D Cell Culture Infection Models

A major limitation in 3D cell cultures is the inability to perform live screening of virus infection, as most 3D models do not permit a direct microscopic view on the infected cells. An additional problem is the usually time-consuming and elaborate preparation of 3D cultures that is needed to process them for microscopic analyses.

**Figure 6:**
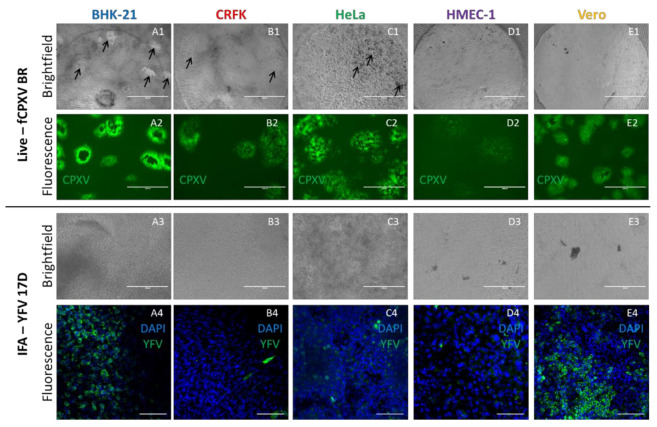
Direct in situ microscopy and horizontal immunofluorescence analyses of infected Wellbricks. Representative pictures of BHK-21 (**A1–A4**), CRFK (**B1–B4**), HeLa (**C1**–**C4**), HMEC-1 (**D1**–**D4**), and Vero (**E1**–**E4**) cells which were cultivated on Wellbricks (10 wt% gelatine, w/o Collagen A) for 3 days followed by infection with CPXV BR for 48 h (1 + 2) and with YFV 17D (3 + 4) for 72 h. Imaging of CPXV infection was performed in situ during cultivation by conventional fluorescence microscopy. CPXV-infected cells were visualised by monitoring GFP expression (green) from recombinant CPXV (**A2**–**E2**). Cytopathic effects induced by CPXV infection seen in the brightfield channel are indicated with black arrows (**A1**–**E1**). For IFA of YFV 17D-infected cells, Wellbricks were fixed and permeabilised en bloc. Infected cells were visualised with a YFV-specific antibody and an AF647 (green)-coupled anti-mouse secondary antibody (**A4**–**E4**). Cellular nuclei were counterstained with DAPI (blue). No cytopathic effects were detected in the brightfield channel (**A3**–**E3**). Analysis of YFV specimens was performed by confocal laser scanning microscopy. Scale bar = 100 µm.

In contrast to previous 3D cultures, the use of the transparent Wellbricks matrix allowed direct immunological staining and IFA of 3D specimens without cryosectioning. As a proof of principle, 3D cell cultures of all cell lines incubated with YFV 17D for 72 h were fixed, permeabilised, and stained with a virus-specific antibody and DAPI. Fluorescence microscopic analyses (Figure 6(A4–E4)) showed that mainly BHK-21 and Vero cells were efficiently infected with YFV 17D which correlated with the analyses of vertical cryosections (Figure 5(E1–E5)).

Another methodological advantage of the Wellbricks-based infection models is the possibility to perform in-situ microscopic analyses of infections without any prior processing of the specimens. Infection of the different cultures with CPXV BR resulted in a cell type-dependent CPE which could be recognised under the microscope in the brightfield channel (Figure 6(A1–E1)) and which was not observable with YFV 17D (Figure 6(A3–E3)). Since the CPXV BR used in these experiments was recombinant for GFP, fluorescence microscopy was used to track the virus spread by following the GFP signal (Figure 6(A2–E2)). The shape and spread of the fluorescent plaques were characteristic for the respective cell type and quite distinct, with the most pronounced plaques detectable for BHK-21 cells and the weakest signals for HMEC-1. The results obtained with this relatively simple method thus correlated with those obtained with the labour-intensive cryosections (cf. Figure 5(A1–A6)).

In addition to the in-situ imaging for the purpose of documenting the spread of infection, the Wellbricks were also used for real-time live cell imaging of infected cultures. Wellbricks with Vero cells were infected with the recombinant CPXV BR (see above) and images were taken at intervals of 20 min for as long as 48 h in both, the brightfield and the GFP channel, in order to follow the formation of a CPE and the distribution of the virus within the cell culture. The resulting videos of the infection process showed a rapid spread of virus infection with an occasional establishment of distinct plaques in the infected cell layer (Supplementary Materials, Video S1).

Taken together, the structure of the Wellbricks allows easy access to different microscopic analyses for virus infection experiments in more in vivo-like 3D cell cultures that is comparable to microscopic analyses of simple, conventional 2D monolayer cultures.

### 3.6. 3D Wellbricks Cultures Are Long-Term viable with Unaltered Permissiveness for Virus Infection

In a critical clinical situation or public health incidence, it might be necessary to rapidly propagate a suspected virus from a diagnostic specimen in cell culture. Under these circumstances, cells should be ready to use because valuable time would be lost if cryo-conserved cells have to be taken first into culture and propagated. Therefore, a system providing long-term cell cultures would be an advantage for cultivation of viruses from urgent clinical specimen.

**Figure 7:**
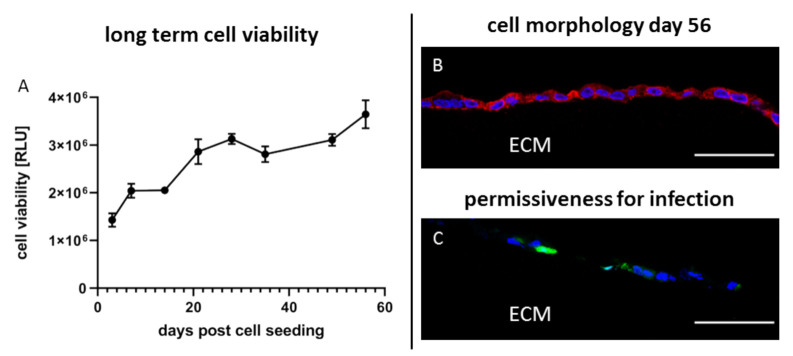
Long-term cultivation of Vero cells on 3D Wellbricks matrices for 8 weeks. Vero E6 cells were cultivated on Wellbricks for 56 days with regular medium exchange every 84 h. Cell viability of uninfected 3D cultures (*n =* 2) was determined once a week by ATP measurement with CellTiter-Glo^®^ (Promega) (**A**). The 8-µm cryosections of Wellbricks were analysed by fluorescence microscopy. To illustrate the unaltered cell appearance, cryosections were prepared on day 56 and cellular nuclei were counterstained with DAPI (blue) and CF594 (red)-coupled wheat germ agglutinin (WGA) for staining of sialic acid and N-acetylglucosamin on cell membranes (**B**). Vero cells on Wellbricks were cultivated for 55 days and then infected with CPXV. Cryosections taken 24 h p.i. were prepared and counterstained with DAPI (blue). CPXV-infected cells were visualised by monitoring GFP expression (green) of recombinant CPXV. Scale bar = 50 µm (**C**).

Since 3D cultured cells display many aspects of the cells in their original tissue and since tissues in general have a much longer life span than conventional 2D monolayer cultures 3D cultures on Wellbricks might be useful for the detection of viruses through cultivation., Therefore, it was examined how long a 3D cell culture can be cultivated without loss of viability. For this purpose, Vero cells were seeded on Wellbricks and continuously cultivated with regular change of growth medium but without any splitting of the culture. Cell morphology and cell viability of the cells were determined at intervals of 7 days. Viability gradually increased up to day 20 post cell seeding and then remained on a constant level until cultivation was ended on day 56 (Figure 7A).

Morphologically, the cells did not change over the entire period of time (see Figure 3E and Figure 7B). Next, it was analysed whether the permissiveness of the cells for virus infection was unchanged after such extended precultivation periods. In this regard, 3D Vero cells precultivated for 55 days were infected with CPXV with an MOI of 0.01 and prepared for IFA 24 h p.i. Analysis of cryosections showed that the permissiveness of the cells for CPXV infection was unchanged compared to a precultivation period of 3 days (see Figure 5(A5) and Figure 7C).

Taken together, this 3D cell culture model can be continuously cultivated over an extended period of time with stable cell characteristics. The culture remains permissive for virus infection without requiring major efforts for its maintenance beyond regular change of medium and thus would be ready to use for spontaneous detection of viruses from suspect samples.

## 4. Discussion

### 4.1. 3D Bioprinted Matrices Promote In Vivo-Like Growth of Different Cell Types

The 3D bioprinted matrices have been designed and optimised to provide a robust, reproducible, and easy-to-handle 3D culture support for cell growth, and have finally been named Wellbricks. Despite the relatively simple biological composition of the matrix, which consists of gelatine and collagen additives, the cell lines used in this study adhered to the support, divided, and developed specific morphological characteristics. This is probably not only related to the composition of the matrix but also to its structure which is very reminiscent of basal laminas of different tissues and other 3D cell culture matrices such as Matrigel in terms of morphology and pore size [39,40]. Therefore, we assume that the cells grew on a surface with an in vivo related micro structure. The five cell lines used cover a large range of commonly used target cells for the cultivation of a broad spectrum of viruses and originate from different cell types, tissues, and species. The epithelial CRFK cells, which originate from the renal cortex, grew on Wellbricks in several layers in a cuboidal (upper layers) to squamous (lower layers) morphology, which can also be observed in the transitional epithelium of the renal pelvis [41]. In contrast, Vero cells, which also originate from the kidney, showed a cuboidal, single-layered cell growth typical of most of the kidney epithelium, e.g., for collecting ducts [42]. The endothelial HMEC-1 cells also grew in single-layered, albeit squamous morphology which resembles the growth of human endothelial cells in blood vessels [43]. Epithelial, cervical HeLa cells grew in stratified, squamous to cuboidal morphology, which can also be observed in the basal and partly endocervical epithelium [44]. Tissue-specific growth was most difficult to define for the fibroblast cell line BHK-21, which in this model was cultivated on and not embedded into the ECM; the latter would correspond to their growth in vivo. However, in contrast to the conventional monolayer, BHK-21 cells grew in a stratified and less flattened manner than in 2D culture, which resembles more closely in vivo behaviour of fibroblasts [45]. Morphologically, the established 3D cell culture models thus still mirror the respective in vivo human tissues more closely than 2D cultures despite the transformational changes that have occurred. In addition, Vero cells exhibited asymmetric protein localisation of typical epithelial polarisation markers on the Wellbricks matrices and were cultivable over a period of 8 weeks without changes in viability, polarisation, and permissiveness for infection. Corresponding monolayer cultures showed none of these in vivo-like characteristics [46].

Although growth characteristics of the cell lines used in this study were hardly affected by the matrix composition, it should be noted that matrix configuration may still have an effect on 3D cell growth and should be established carefully. We observed that even small changes in the gelatine concentration of the Wellbricks from 10 to 7 wt%—and thus in the matrix structure and pore size—already caused a heavy loss of cell viability and a considerable morphological change of primary keratinocytes in another study (data not published). Similar dependencies of the cell growth on the pore size of different hydrogels could also be observed in other studies; however, results were often contradictory and strongly dependent on the respective cell type [47,48,49]. Two studies which investigated comparable approaches of cell cultivation showed that for successful growth of cells on a matrix the pore size of the matrix must not deviate from the pore size of a natural basal lamina of about 20–125 nm [50,51,52]. These data are consistent with our results.

### 4.2. Wellbricks 3D Cell Cultures Are Suitable for the In Vivo-Relevant Investigation of Viral Infections

In order to investigate the versatility of this 3D cell culture model for infection experiments, we infected the established 3D cell cultures with different viruses. Although this has not been designed as a comprehensive study, we tried to cover a broad spectrum of different viral pathogens. RNA and DNA viruses of large and small size as well as enveloped and nonenveloped viruses were included. The investigated viruses also differed in their replication rate, the virus load per cell, their host range, their cytopathogenicity, and their species specificity (Table A3). Furthermore, the viruses are low pathogenic surrogate viruses of highly pathogenic viruses with public health relevance. Therefore, the infection models established here represent a meaningful proof of concept for further infection models that can be established based on this study.

Virus detection in the 3D cell culture models was successful both on the molecular and on the morphological level. Immunofluorescence analyses of histologically processed specimens for visualisation of virus proteins and virus-mediated changes in cellular protein biosynthesis could be reproducibly performed because of the good maintenance of matrix integrity of the formaldehyde-fixed cryosections. As a special feature, the transparent matrices also enabled to track possible virus-induced changes of cell morphology in real time, thus allowing a rapid estimation of the viral replication rate and cytopathogenicity. Due to the higher biological complexity of 3D cell culture models, information on cell tropism, host range, or cytopathogenicity might be more reliable and more relevant than information generated in a conventional 2D cell culture. The combination of the mentioned advantages of the infection models on the physiological and methodological level and the potential of the Wellbricks matrices to be suitable for the cultivation of many other cells—and thus propagation of a broad range of viruses—enables a comprehensive characterisation of virus–host interactions under in vivo-like conditions. This also includes viruses such as HAV, HuNoV, and HPV which cannot be propagated in conventional 2D cell cultures because of their requirements for host cell differentiation or their low replication rate [3,4,11].

### 4.3. Wellbricks 3D Cell Cultures Are Superior to Other 3D Cell Culture Models in Terms of Handling and Infection Screening

Compared to other 3D cell culture infection models developed for a broad spectrum of different viral pathogens [5], the Wellbricks infection models established in this study offer crucial advantages. This is mainly due to the highly reproducible and automated production process for the matrices. While most cell culture models are assembled under the laminar flow by manual sequences [53], Wellbricks are produced by 3D bioprinting and therefore can be designed with a high degree of flexibility and be produced in a highly defined manner [21,24]. Therefore, the appearance and structure of the matrices can be adapted to the requirements of the respective labs. For example, the matrices can be printed with or without cultivation barriers and in different forms and sizes regarding growth area or matrix thickness. Additionally, the composition of the matrix is fully definable and their biological components can be adapted to the requirements of the cells or even viruses, which may differ greatly from each other [53,54]. Besides variability of biological components like different collagens or elements of the basal lamina even viable cells such as fibroblasts or other mesenchymal cells, which represent an essential part of biological ECMs, could also be printed directly into the matrix [27]. Due to rapid seeding and the ease of cell cultivation, matrix composition can be tested rapidly for compatibility with different cells and thus be optimised rapidly through an iterative process until achieving a suitable configuration.

Compared to other 3D cell culture models, another significant advantage is the high reproducibility of the printed matrix and thus the reproducibility of the 3D cell cultures. With the exception of simple spheroid cultures, the lack of reproducibility is one of the main shortcomings of most 3D cell cultures, which also affects their acceptance in the scientific community [55]. Further, the quality of cell growth on Wellbricks can be verified in situ by standard microscopy because of their transparency. This fast and simple optical examination of the 3D cell culture is a considerable advantage over the majority of other 3D cultures where cells can only be visualised using complex microscopy methods such as multi-photon microscopy or optical coherence tomography [56].

### 4.4. Wellbricks 3D Cell Cultures Represent a Suitable Tool for Virus Detection

Virus cultivation is an important and powerful tool in virus diagnostics. However, to establish a new cell culture approach as a diagnostic tool that is superior to conventional virus cultivation techniques it will have to fulfil certain requirements for the propagation and initial characterisation of viral pathogens. Such a tool should be highly reproducible, ready for use as quickly as possible, usable for a large panel of different viruses, and ideally be easy to handle [57]. For the established Wellbricks infection models, the reproducibility of cell growth and the handling of the preparations are comparable to those of conventional 2D cultures. In contrast to 2D cultures, the 3D specimens can be cultivated for more than 8 weeks without splitting the cells, and without changing their growth behaviour or their permissiveness to infection. Thus, on Wellbricks, the 3D cultures can be prepared and kept in culture for extended periods of time ready-to-use for infection with patient samples without precultivation of cells. On the other hand, because of the extended cultivation time the 3D cultures facilitate propagation of slowly replicating viruses which might be lost in conventional 2D cultures since it is necessary to split the cell cultures regularly with the risk of losing the virus [58]. The 3D in vivo-like growth of the cells cultured on Wellbricks also has the potential to support replication and maturation of viruses such as HPV, HuNoV, and HEV, as already shown for other 3D cultures [8,13,59]. Furthermore, the biologically more relevant virus–host interactions in a 3D cell culture may help estimate rapidly the host range and tissue tropism of a virus. Finally, this 3D cell culture system using printed matrices can easily be adapted to other cells. Therefore, it has the potential to establish cell culture models that are relevant for an in vivo-like investigation of a broad spectrum of human and animal viral pathogens because of their definable and modifiable matrix composition and structure, including the possibility of imprinting additional cell types for cocultivation of cells [27].

In summary, we established 3D cell culture models for five different cell lines based on the bioprinted Wellbricks matrix. The cells in these 3D cultures differ significantly from conventional monolayer cell cultures and mirror the respective in vivo tissue regarding cell morphology, polarity, and long-term stability. Due to the transparency of the Wellbricks matrix the established infection models enable an easy screening of virus spreading by microscopy. Furthermore, the models allow a first in vivo-like characterisation of virus infection regarding cytopathogenicity and host range because of the 3D tissue-like cell growth. However, there is still no proof that the models are actually permissive for viruses that cannot be propagated in conventional 2D cultures. In addition, the possibility to estimate tissue tropism and host range of viruses via characterisation with these models more realistically than with 2D cultures has to be verified in subsequent studies and remains an assumption so far.

In order to implement the models for routine diagnostics, they should be further simplified. We currently work on combining individual cultures in an array format in which cells can be cocultivated in one well of a cell culture plate. Thus, inoculation of a single array with a patient sample would provide a broad range of potential host cells, which will significantly simplify screening for a suitable virus cultivation system for the propagation and first characterisation of the pathogens.

## Supplementary Materials

The following are available online at https://www.mdpi.com/1999-4915/12/11/1298/s1, Video S1: Live-cell imaging of CPXV infected Vero 3D cultures on Wellbricks illustrates fast virus dissemination and formation of CPE. Vero E6 cells were cultivated on Wellbricks for 3 days before infection with GFP-recombinant CPXV. Infection was monitored in a cultivation chamber of an epifluorescence microscope under standard cultivation conditions for 48 h. Images in the brightfield and the GFP channel were taken every 20 min. Shown here is the period between 3 and 35 h after onset of the measurement. CPXV infected cells were visualised by monitoring GFP expression (green) from recombinant CPXV strain. Scale bar = 100 µm.

## Appendix A

**Table A1:** Characteristics of applied eukaryotic cell lines.

Cell Line	Species	Tissue	Cell Type	Origin *
BHK-21	*Mesocricetus auratus*	Kidney	Fibroblast	ATCC; #CCL-10
CRFK	*Felis catus*	Kidney	Epithelial	Friedrich Loeffler Institute; CCLV-RIE 769
HeLa	*Homo sapiens*	Cervix	Epithelial	ATCC; #CCL-2
HMEC-1	*Homo sapiens*	Dermal	Endothelial	ATCC; #CRL-3243
Vero E6	*Chlorocebus sabaeus*	Kidney	Epithelial	ECACC; #85020206

* ATCC: American Type Culture Collection; CCLV-RIE: Collection of Cell Lines in Veterinary Medicine; ECACC: European Collection of Authenticated Cell Cultures.

**Table A2:** Purification conditions of the applied viruses.

Virus *	Purification Buffer	Sucrose Cushion	Speed (× *g*)	Time (h)
FCV	10 mM TrisHCl	30% sucrose	71,100	5
MVA BN	10 mM TrisHCl	36% sucrose	32,900	1.3
PUUV Kazan	TNE buffer	30% sucrose	153,700	2
YFV 17D	10 mM TrisHCl	36% sucrose	153,700	5

* FCV: *Feline calicivirus*; MVA BN: *Modified Vaccinia Ankara* – Bavarian Nordic; PUUV Kazan: *Puumala virus*; YFV 17D: *Yellow fever virus* 17D vaccine strain.

**Table A3:** Characteristics of viruses based on experimental data.

Feature/Virus	CPXV BR	MVA	FCV	PUUV	YFV 17D
Nucleic acid	dsDNA	dsDNA	ss(+)RNA	ss(−)RNA	ss(+)RNA
Replication rate (in vitro)	High	Intermediate	High	Low	Low
Virus genomes per cell (in vitro)	Intermediate	Intermediate	High	Low	High
Known host range (in vitro)	Broad	Intermediate	Narrow	Narrow	Intermediate
Cytopathogenic (in vitro)	Yes	Yes	Yes	No	No
Human pathogenic	Yes [60]	No [61]	No [62]	Yes [63]	No [64]
Surrogate for *	MPXV, VARV [65]	(MPXV, VARV)	NoV [66]	DOBV/HTNV [67]	YFV [64]

* MPXV: *Monkeypox virus*; VARV: *Variola virus*; NoV: *Norovirus*; DOBV: *Dobrava-Belgrade virus*; HTNV: *Hantaan virus*; YFV: Y*ellow Fever virus*.
